# Anti-Doping Knowledge of Students Undertaking Bachelor’s Degrees in Sports Sciences in Spain

**DOI:** 10.3390/nu14214523

**Published:** 2022-10-27

**Authors:** Millán Aguilar-Navarro, José-Antonio Salas-Montoro, José Pino-Ortega, Juan José Salinero, Fernando González-Mohíno, Virginia Alcaraz-Rodríguez, Diego Moreno-Pérez, Nadia Lanza, Beatriz Lara, Víctor Moreno-Pérez, Blanca Romero-Moraleda, Alberto Pérez-López, Carlos García-Martí, Juan Del Coso

**Affiliations:** 1 Exercise and Sports Sciences, Faculty of Health Sciences, Universidad Francisco de Vitoria, 28223 Madrid, Spain; 2 Department of Physical Education and Sport, Faculty of Sports Sciences, University of Granada, 18071 Granada, Spain; 3 BioVetMed & SportSci Research Group, University of Murcia, 30100 Murcia, Spain; 4 Sport Training Laboratory, Faculty of Sports Sciences, University of Castilla la Mancha, 45071 Toledo, Spain; 5 Facultad de Ciencias de la Vida y de la Naturaleza, Universidad Nebrija, 28240 Madrid, Spain; 6 Faculty of Education, Universidad Internacional de Valencia, 46002 Valencia, Spain; 7 Department of Education, Research and Evaluation Methods, Comillas Pontifical University, 28049 Madrid, Spain; 8 Faculty of Education and Sport, University of Deusto, 48007 Bilbao, Spain; 9 Exercise Physiology Laboratory, Camilo José Cela University, 28692 Villanueva de la Cañada, Spain; 10 Center for Translational Research in Physiothearapy, Department of Pathology and Surgery, Universidad Miguel Hernández, 03202 Elche, Spain; 11 Department of Physical Education, Sport and Human Movement, Autonomous University of Madrid, 28049 Madrid, Spain; 12 Departamento de Ciencias Biomédicas, Área de Educación Física y Deportiva, Facultad de Medicina y Ciencias de la Salud, Universidad de Alcalá, 28871 Madrid, Spain; 13 Faculty of Sport Sciences, Universidad Europea de Madrid, 28670 Villaviciosa de Odón, Spain; 14 Centre for Sport Studies, Rey Juan Carlos University, 28943 Fuenlabrada, Spain

**Keywords:** sports performance, sports nutrition, physical activity, performance enhancement, education, university

## Abstract

In Spain, students pursuing a career in athletic training, physical education, or scientific evaluation of sports enroll in a bachelor’s degree in sports sciences. This degree provides knowledge and skills in a broad array of sports settings and promotes research-based interdisciplinary knowledge. However, the student’s syllabus rarely includes specific academic training on anti-doping regulations or doping prevention. The purpose of this study was to assess the anti-doping knowledge of the students undertaking a bachelor’s degree in sports sciences in Spanish universities. One thousand two hundred and thirty-three bachelor students in sport science (907 males, 322 females, and 4 participants with non-binary sex) from 26 Spanish universities completed a validated questionnaire about general anti-doping knowledge. The questionnaire is an adapted version of the Play True Quiz of the World Anti-Doping Agency and contains 37 multiple-choice questions. The score obtained in the questionnaire was transformed into a 0–100-point scale. The questionnaire was distributed among students within each university by a faculty member and it was filled out online. Students obtained a score of 65.8 ± 10.10 points (range = 32–92 points). There was an effect of the course in the score obtained (*p* < 0.001). Students of the first course (63.6 ± 9.5 points) had lower scores than the remaining courses (*p* < 0.037) while the students of the fourth course obtained the highest scores (68.7 ± 9.5 points; *p* < 0.019). The students with an itinerary on sports performance were the respondents with the highest anti-doping knowledge (67.2 ± 10.2) points, followed by the students with an itinerary on health (66.7 ± 9.5 points). The knowledge of basic anti-doping rules and doping prevention strategies of the bachelor students in sports sciences in Spain was suboptimal. Increasing doping prevention information in the syllabus of the bachelor’s degree in sports sciences is essential as these future professionals will directly work with populations at risk of doping.

## 1. Introduction

Most sports have a competitive nature and athletes want to win, or at least they wish to perform at their best. Sports training is one of the most important factors that athletes use to improve their physical and mental performance although it must be programmed in the long term to provide significant enhancements. Sports nutrition can contribute to optimizing physical and physiological adaptations obtained through training. Sports nutrition aids sports performance through proper caloric, nutrient, and hydration intake during training and competition, although the exact amount of nutrients needed and the timing to ingest them are highly dependent on the characteristics of each sport [1]. In addition to diet, athletes use dietary supplements because they provide a more convenient form of energy, or to increase the amount of certain substances that cannot be properly obtained with the diet [2]. The employment of supplements is widespread at all levels of sport, and athletes and sports practitioners (i.e., athlete support personnel) must be aware of the potential benefits and drawbacks of dietary supplements. The knowledge of pros and cons of dietary supplements is essential because a high proportion of supplements available in the market are not effective [3], are not innocuous, or are contaminated with prohibited substances [4]. For the athlete support personnel, protection of the athlete’s health must be paramount, and they must be vigilant to avoid that athletes use—either intentionally or inadvertently—prohibited substances. Collectively, athlete support personnel should have appropriate anti-doping knowledge to ensure athletes remain safe and clean [5].

The fight against doping is a challenging task that requires a multidisciplinary approach including medical, psychological, and sociological measures to be successful. This is because the vulnerability to doping spreads beyond the elite athlete population and reaches other groups of athletes and sportspeople [6], including amateur athletes and adolescents [7,8]. Although the main anti-doping actions have been historically based on the detection of prohibited substances in bodily samples, the complexity and reach of modern doping require that prevention measures conform to the core component of any anti-doping program. The World Anti-Doping Agency (WADA) has wide a range of educational initiatives to support the anti-doping community and complement the deterrence power of doping controls. Among others, increasing access to education for athletes through a global and local education network is one of the main activities of WADA. The importance of education as a key aspect of the fight against doping has been recently certified by the implementation of the International Standard for Education as part of the changes introduced in the last update of The Code in 2021 [9]. Most of the education-based programs of WADA and other national anti-doping authorities have been focused on delivering activities that emphasize the development of an athlete’s values and principles toward a clean sport. For example, the Italian Federation of Athletics promoted the *Lotta al Doping* (Fight Against doping) program, an initiative that was effective to produce a cultural shift in students through anti-doping seminars at high schools [10]. However, most of these initiatives are focused on the athlete’s behaviors and environment [11]. Effective anti-doping education programs must specifically target athlete support personnel, as athletes habitually rely on them when planning the use of supplements and ergogenic aids [3].

According to The Code [9], athlete support personnel include coaches, doctors and other medical personnel, parents, along with anyone else who assists athletes in their competitive life. They have a vital role to prepare the athlete for competition but also have the responsibility of ensuring that athletes fulfill their responsibility to support a clean sport. However, the fight against doping encompasses a wide variety of aspects that are beyond the knowledge of many sports coaches [12,13]. To date, although WADA education initiatives reach athlete support personnel, coaches must be empowered to meet the challenge of preventing doping during their formation career.

In Spain, students pursuing a career in athletic training, physical education, or scientific evaluation of sports enroll in a bachelor’s degree in sports sciences. This degree provides knowledge and skills in a broad array of sports settings and promotes research-based interdisciplinary knowledge. Among other aspects, students are trained to plan, organize, regulate, direct, and evaluate the different practices related to physical activity and sports. The degree includes a common basic formation and then students can specialize in a specific itinerary through the selection of optional subjects. However, the student’s syllabus rarely includes specific academic training on anti-doping regulations or doping prevention. The implementation of an anti-doping education in university students may be key to producing exercise practitioners with lifelong anti-doping behaviors as prevention through education programs is more effective in young individuals when attitudes and values are being formed and linked to social and skill development [14]. To this respect, efficacious anti-doping interventions in school settings have been effective to produce changes in the attitudes toward doping in a large audience of young people [10,15], although the efficacy of education programs is rarely studied in universities. Despite sports science university students being expressly trained to become supporting personnel for athletes, the current scenario may induce a suboptimal preparation to prevent doping due to the lack of anti-doping education in the university. The purpose of this study was to assess the anti-doping knowledge of bachelor students undertaking sports sciences degrees in Spanish universities.

## 2. Materials and Methods

### 2.1. Participants

One thousand two hundred and ninety-eight students volunteered to participate in this investigation by filling out a validated and reliable questionnaire about anti-doping knowledge. All participants had to be active students undertaking a bachelor’s degree in sports sciences at a Spanish university. Participants were recruited from 26 different universities with analogous characteristics and contents (4 years with 240 credits of the European Credit Transfer and Accumulation System) for the bachelor’s degree, as the syllabus was designed following the guidelines of the National Agency for Quality Assessment and Accreditation of Spain. Taking part in the study was voluntary and participants gave their consent to participate by accepting a clause included at the beginning of the questionnaire. No personal information that allows the identification of the participants was obtained during the study. Sixty-five students were excluded from the study because they did not complete the questionnaire, were not university students, or were studying for other bachelor’s degrees. Additionally, six questionnaires were not considered valid because they contained duplicate information. Hence, the final sample was composed of 1233 bachelor students undertaking a degree in sports sciences. The questionnaire was distributed to 3196 students and the response rate was 39.2% (ranging from 7.8% to 60.3% in the different universities participating in the study). The procedures developed in this investigation were approved by an institutional Ethics Review Committee and the study was carried out in accordance with the procedures approved by the Declaration of Helsinki. Specific information about the study sample can be found in Table 1.

### 2.2. Questionnaire and Data Collection

This study was developed following the Checklist for Reporting Results of Internet E-Surveys (CHERRIES) [16]. The data collection was carried out between September 2021 and December 2021. The questionnaire was presented by a faculty member of each participating university during one of the classes, who explained the objective of the study and explained the characteristics of the questionnaire. The faculty member indicated that the questionnaire did not collect identifiable information from the respondent and that participation in the study was voluntary with no positive or negative implications on the marks of the students. Additionally, no other incentive was offered for those participants willing to participate. Once participants accepted the invitation to partake in the study, the faculty member provided a web link, and students started the questionnaire in their personal laptop or tablet. This process indicates that the questionnaire can be considered as a “closed” survey as the access was only available for those willing to participate. As the questionnaire assesses anti-doping knowledge, participants had to fill out the questionnaire on their own without consulting any other document or webpage, which was verified by the faculty member. The faculty member solved any questions that were not associated with the anti-doping contents of the questionnaire. Participants had no time limit to complete the questionnaire, although all participants completed the questionnaire between 15 and 39 min. Once the questionnaire was completed, participants obtained information about the score they obtained. Once all participants had completed the questionnaire, the link was disabled to avoid participants from completing the questionnaire in a context different from the one explained above. The implementation of the survey was limited to universities with official bachelor’s degrees in sports sciences in Spain.

The questionnaire used in this research was an adapted version of the “WADA Play True Quiz” [17]. This questionnaire contains multiple true/false and multiple-choice questions about basic anti-doping knowledge and was developed by WADA as an interactive computer game that tests athletes’ knowledge about anti-doping in 45 different languages. An adapted version of the questionnaire was created by two sports scientists (MAN and JDC) with experience in anti-doping (Appendix A). The adapted version was developed—instead of the use of the original version—for three reasons: (a) In the original version, participants are addressed as athletes; (b) there are ten questions that inquire about behaviors/attitudes toward doping rather than anti-doping knowledge; and (c) there are no questions associated with sociodemographic variables. Hence, the adapted version of the questionnaire opens with a section of sociodemographic questions about the participant’s gender, university, course, itinerary of studies selected in sports sciences degrees (sports performance, health, sports management, physical education, other, none), registry in a sports federation, and if they are using dietary supplements. Then, the section about anti-doping knowledge is presented which includes questions formulated for participants who are athlete supporting personnel and 27 questions of the original version, plus 8 new questions about anti-doping knowledge, for a total of 37 questions (Appendix A). In all questions, participants had to select the correct response from a list of potential answers, but they did not have to enter any information with a keyboard to respond. The order of the questions was not randomized, and participants had visual access to a toolbar on the screen that indicated the number of questions responded to, and the number of questions still to be filled. During the filling process, participants had access to “back” and “forward” to review and change their answers, if necessary. The technique used for the distribution of the questionnaire did not allow for the identification of IP addresses or cookies. However, participants were encouraged to fill out the questionnaire only once and the link containing the questionnaire was disabled once participants in one class had finished the questionnaire.

A one-point value was given to each question associated with anti-doping knowledge for a maximal score of 37 points. Then, the score was transformed into a 0–100-point scale to facilitate the comprehensibility of the level of anti-doping knowledge demonstrated with the questionnaire. The construct validity of this adapted version of the WADA Play True Quiz was verified by a panel of four anti-doping experts working for the National Spanish Anti-Doping Agency following previous recommendations [18]. The evaluation of the construct validity of the questionnaire to assess anti-doping knowledge was carried out individually by each expert and there was no contact among them to avoid any interference in this process. On the final form of the questionnaire, all the questions were introduced in an online form of Google (Mountain View, CA) to facilitate the distribution and the data collection. The reproducibility of the score obtained in the adapted version of the WADA Play True Quiz was evaluated in a group of 56 students from two different Spanish universities. Participants completed the adapted version of the questionnaire on two occasions separated by four weeks, as previously recommended [19]. The conditions under which the questionnaires were completed in the test–retest were maintained to avoid the influence of extraneous variables on the results of the reproducibility [20]. Participants had the possibility of completing a specific section available only for the test–retest about any difficulty experimented during the filling of the questionnaire or regarding the comprehensibility of any question. There were no significant differences between the test and the retest (test score: 65.68 ± 8.92 points vs. retest score: 67.57 ± 9.46 points; *p* = 0.43); coefficient of variation: 3.6%; intraclass correlation coefficient: 0.77). Additionally, a previous study using a 15-item version of the WADA Play True Quiz reported high values of internal consistency [10].

### 2.3. Statistical Analysis

After the data collection, data were organized, checked, and analyzed with the statistical package SPSS 27 (SPSS Inc., Chicago, IL, USA). The number of participants in the sociodemographic variables is expressed by frequencies and percentages. The normality of scores obtained in the questionnaire was initially tested with the Kolmogorov–Smirnov test. This variable was normally distributed for the whole sample and for the groups created to perform comparisons. Hence, parametric statistics were used to determine differences between/among groups. When comparing two groups (students had/had not received anti-doping lessons, students were/were not registered in a national sports federation, students did/did not report the use of supplements, students practicing individual/team sports) the differences in the score between groups were identified by using unpaired *t*-tests. When comparing more than three or more groups (e.g., student’s course and itinerary), the differences in the score among groups were identified using a one-way analysis of variance (ANOVA). In the case of an F-significant test, the Tukey post hoc test was applied to detect differences in the pairwise comparisons of the groups. The score obtained in the questionnaire is expressed as mean ± standard deviation. The significance level was set at *p* < 0.050.

## 3. Results

**Figure 1:**
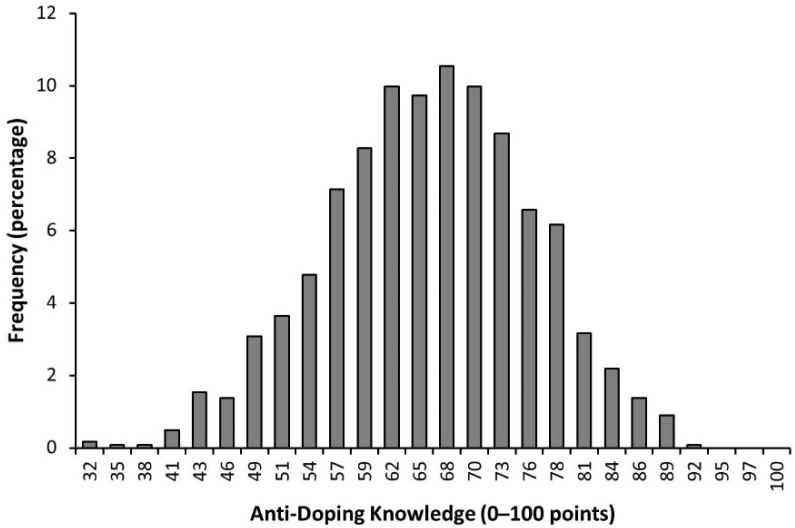
Frequency of bachelor students undertaking a degree in sports sciences in Spanish universities according to their anti-doping knowledge measured by an adapted version of the WADA Play True Quiz. Each bar represents the percentage of students, from a total of 1233, who obtained a given score in the questionnaire.

On average, the students obtained a score of 65.78 ± 10.11 points (range = 32–92 points). From the total, 6.8% of students obtained a score below 50 points and 85.5% of students obtained a score between 50 and 80 points. Only 0.1% of the respondents obtained a score above 90 points (Figure 1).

**Figure 2:**
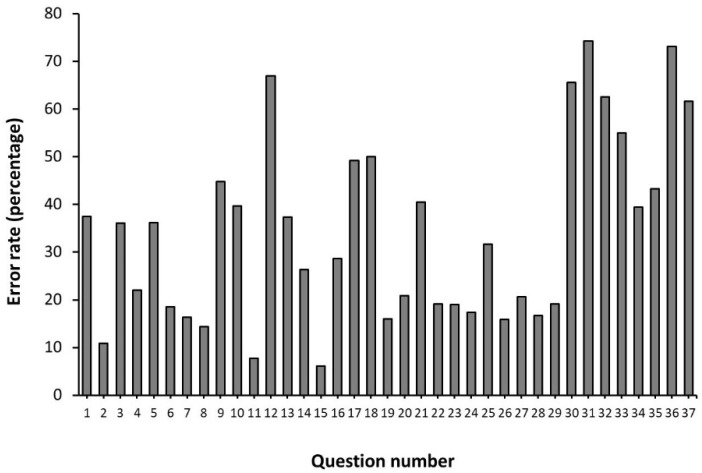
Rate of error in the questions of the adapted version of the WADA Play True Quiz in Spanish bachelor students undertaking a degree in sports sciences. Each bar represents the percentage of students, from a total of 1233, who chose a wrong answer for each of the 37 questions of the questionnaire.

Question number 15, associated with the meaning of the acronym TUE, which refers to therapeutic use exemption, was the question with the lowest rate of error (6.1% of error, Figure 2). Question number 31, associated with the different scenarios that can end in an anti-doping rule violation, was the question with the highest rate of error (74.3%). In total, there were eight questions in which the rate of error was superior to 50% of the sample.

**Figure 3:**
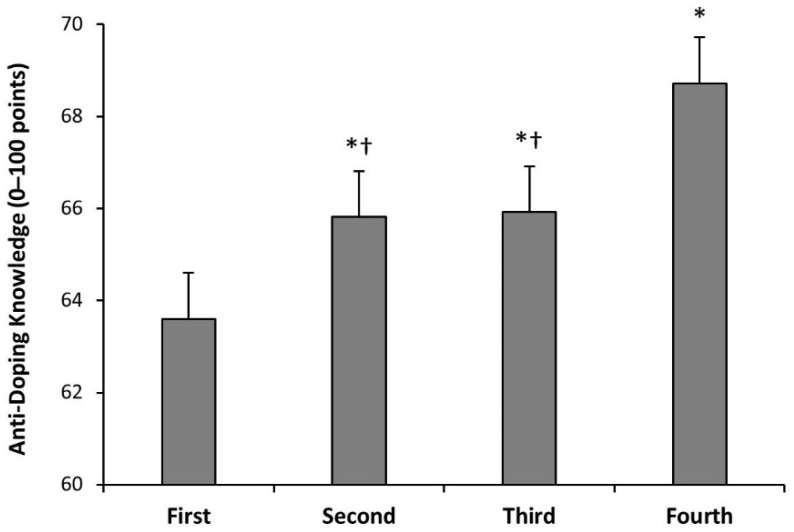
Scores in the adapted version of the WADA Play True Quiz of students undertaking a degree in sports sciences in Spanish universities according to their course. Data for each course are presented as mean ± standard deviation. (*) Different from students in the first course at *p* < 0.050, identified with Tukey post hoc. (†) Different from students in the fourth course at *p* < 0.050, identified with Tukey post hoc.

**Figure 4:**
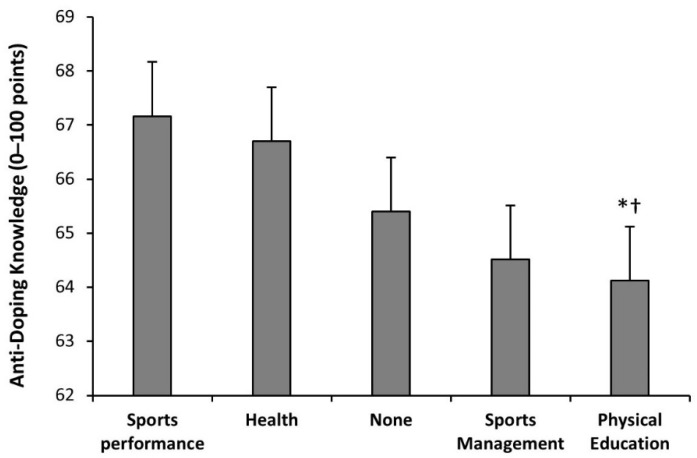
Score in the adapted version of the WADA Play True Quiz of bachelor students undertaking a degree in sports sciences at Spanish universities according to their itinerary. Data for each itinerary are presented as mean ± standard deviation (*) Different from students in the itinerary of sports performance at *p* < 0.050, identified with Tukey post hoc. (†) Different from students in the itinerary of health at *p* < 0.050, identified with Tukey post hoc.

There was an effect of the course on the score obtained in the anti-doping knowledge questionnaire (F = 9.43; *p* < 0.001). Bachelor students enrolled in the first course of the degree had lower scores than the remaining courses (*p* = 0.037). The students of the second and third courses had similar scores, but their scores were inferior to students of the fourth course (*p* = 0.019; Figure 3). There was an effect of the itinerary on the score obtained in the anti-doping knowledge questionnaire (F = 5.97; *p* < 0.001). Bachelor students with an itinerary on sports performance were the students with the highest anti-doping knowledge, followed by the students with an itinerary in health (Figure 4). The students with a physical education itinerary had the lowest scores and they were different from the students with a sports performance itinerary (*p* = 0.001) and students with a health itinerary (*p* = 0.003).

From the total sample, only 285 (23.1%) students had received anti-doping information/lessons in any of the subjects of the degree. The score obtained by students that manifested in receiving anti-doping information/lessons obtained a similar score to the students who had not received anti-doping lessons during the degree (65.82 ± 10.47 and 65.77 ± 10.01 points, respectively; *p* = 0.940). Seven hundred and sixty-eight students (62.3%) were registered in a national sports federation. The score in anti-doping knowledge was higher in the students registered in sports federations than in the students not registered in any sports federation (66.27 ± 9.88 vs. 64.98 ± 10.43 points, respectively; *p* = 0.028).

There were 426 (34.5%) students that reported the use of dietary supplements at the time of the questionnaire filling. Those students using supplements had higher anti-doping knowledge scores than their counterparts who did not use dietary supplements (67.80 ± 10.81 vs. 64.88 ± 9.60 points, respectively; *p* < 0.001). In the sample, 1152 (93.4%) students practiced any form of exercise or sport at least three days per week while the remaining 5.4% did not reach this threshold of exercise frequency. Among the students that practiced sport, there were a total of 24 sports modalities. The students practicing individual sports obtained a higher score in the anti-doping knowledge questionnaire than the students practicing team sports (66.74 ± 10.47 vs. 64.80 ± 9.57 points, respectively; *p* < 0.001).

## 4. Discussion

The aim of this study was to assess the anti-doping knowledge of students undertaking a bachelor’s degree in sports sciences at Spanish universities. We pursued this aim because the students’ syllabus of this degree rarely includes specific academic training on anti-doping regulations or doping prevention, despite the degree offering qualifications to work with athletes at all levels of performance. To fulfill this aim, we used an adapted version of the WADA Play True Quiz, and we gathered anti-doping knowledge from students of 26 different Spanish universities. The main results of this investigation indicate that Spanish students undertaking a bachelor’s degree in sports sciences had a modest knowledge of anti-doping prevention measures and regulations, as the mean score obtained was 65.78 ± 10.11 points (range = 32–92 points), despite the questionnaire inquiring about basic anti-doping knowledge. Although the data showed a progressive increase in the anti-doping score across the courses of the bachelor’s degree, the higher scores in participants registered in a sports federation and those taking supplements suggest that a portion of the anti-doping knowledge of students comes from other sources independent of the university.

Although WADA educational initiatives target all populations of athletes, the anti-doping education programs mainly reach international-level athletes [11] with a lower influence on amateur athletes and athlete support personnel up to now [21]. Current anti-doping education programs prioritize value-based education in sports and the content is focused on preventive approaches rather than on medical reasons for not doping. During the implementation of The Code in 2015, WADA established an e-learning education program, “Athlete Learning Program about Health and Anti-doping” available on the Internet for athletes and support personnel at all competition levels. This program has been enhanced in 2021 with the implementation of the latest version of The Code and the name of the education program has changed to “Anti-Doping Education and Learning platform” (ADEL) [22]. This new education platform features a wider range of educational courses and resources for more target audiences, including athletes, coaches, parents, and medical professionals. Despite these efforts of WADA to implement different education programs and other anti-doping initiatives, the knowledge provided in these programs rarely reaches the sports community beyond elite populations [11]. This is somewhat expected, as it is unfeasible that WADA’s initiatives (or those of national anti-doping organizations) reach to the whole sports community. For this reason, other layers of society must play an active role in the distribution of preventive and educative measures against doping in sports, such as the university.

Previous investigations have assessed the anti-doping knowledge of different athlete support personnel [12,13,23,24]. Despite the different populations tested (coaches, doctors, pharmacists, family, etc.), the different questionnaires used to assess anti-doping knowledge (Athlete Learning Program about Health, anti-doping ALPHA test, Performance Enhancement Attitude Scale, ad hoc questionnaire, and focus group discussion) all investigations coincide in that the anti-doping knowledge of the athlete supporting personnel was moderate and they habitually lacked the skills required to provide athletes with accurate information about anti-doping issues [12,13,23,24]. To the authors’ knowledge, only one investigation has tested the anti-doping knowledge of university students undertaking physical education and sport science-related degree program in Kenya [25]. In that study, participants in the first year of the degree showed less anti-doping knowledge than their counterparts in the fourth course, although the average knowledge of anti-doping policies was modest. Additionally, previous participation in sports also increased anti-doping knowledge in these university students. The current results coincide with these previous investigations because the level of anti-doping knowledge of students undertaking a bachelor’s degree in sports science in Spain was suboptimal. The anti-doping knowledge reflected by the students was suboptimal because, on average, the score obtained was 65.78 ± 10.11 points (over a maximum of 100 points) and only 0.1% of the participants obtained a score above 90 points. These scores should be considered modest because the modified version of the WADA Play True Quiz used in this study included only basic and simple questions about anti-doping regulations and preventive measures. As a comparison, high school students obtained high rates of correct answers in 13 out of 15 items of a modified version of the WADA Play True Quiz upon the completion of a 2 h seminar in anti-doping knowledge [10]. Additionally, the previous questionnaire developed by WADA to assess anti-doping knowledge in athletes and supporting personnel, the ALPHA test, issued a certificate when the rate of correct answers surpassed 80% [11]. Although there are significant differences between the ALPHA test and the WADA Play True Quiz, such as the number of questions (12 vs. 39, respectively), if we apply the same rate in our study, only 9.9% of students would surpass the 80% of correct answers. Overall, the current results suggest that Spanish universities including bachelor’s degrees in sports sciences need to include theoretical and practical lessons of anti-doping knowledge in their curricula to enhance the preparation of their students in the fight against doping when they become professionals.

Since 2013, the Spanish anti-doping agency has promoted several initiatives of education and learning for students undertaking a bachelor’s degree in sports sciences [26]. However, these courses are offered in a limited number of Spanish universities and do not reach a wide proportion of students because participation is voluntary. While this initiative is in line with the latest version of The Code [9] and with the International Standard for Education [27], it is probably insufficient to produce well-educated sports practitioners. In fact, the data of the current investigation indicate that the students that had received anti-doping information/lessons obtained a similar anti-doping knowledge score to those students who had not received anti-doping information during the degree. This indicates that current efforts made to include anti-doping lessons in bachelor’s degrees in sports sciences at Spanish universities are not sufficient to improve the preparation of future sports scientists, probably because these lessons are short (habitually less than 30 h) and optative. Although it has been suggested that universities with degrees in physical education and sports sciences need to partner with anti-doping agencies to increase the anti-doping knowledge of their students [25], the inclusion of an anti-doping subject (compulsory and with at least 60 h of duration) within the syllabus of the degree may be the best approach to obtain sports practitioners with enough knowledge and skills to effectively fight against doping. This is especially important for university students of sports sciences in Spain, as 23.6% of them may be willing to use banned substances and methods to increase their economic revenues as athletes [28].

In total, there were eight questions in which the rate of error was superior to 50% (Figure 2). Only a small percentage of respondents (25.7%) knew the different scenarios that can end in an anti-doping rule violation beyond the presence of a banned substance in a urine or blood sample. Additionally, only 26.8% of respondents knew the difference between a banned substance and an ergogenic aid, and only 33.0% of respondents identified that the WADA list of banned substances and methods applies to all sports. Other questions with high rates of error were associated with the recognition of the authorities that certify a TUE or identifying the purpose of the Athlete Biological Passport. On the contrary, a high portion of students identified that refusing to submit to doping control can carry the same sanction as a positive test (92.2%), that athletes beyond the ones competing in the Olympic Games and World Championships can be submitted to a doping control test (89.1%) and that several anti-doping rule violations apply to coaches, doctor, and other members of the athlete support personnel (85.6%). Collectively, although bachelor students undertaking degrees in sports sciences had high rates of correct responses for some sections of the questionnaire, they still presented extraordinary error rates in questions regarding the therapeutic use exemption and Athlete Biological Passport despite these being key factors of the fight against doping laid out in The Code.

The course in which the students were enrolled was a variable with great influence on anti-doping knowledge (Figure 3). Bachelor students in the first course had lower scores than those in the remaining courses. At the same time, students in the fourth course obtained scores statistically significantly higher than the remaining courses. Interestingly, the itinerary selected during the degree was another predictor of anti-doping knowledge (Figure 4). The bachelor students with an itinerary on sports performance were the students with the highest anti-doping knowledge, followed by students with an itinerary in health. These results indicate that university students progress in their anti-doping knowledge across the four courses of bachelor’s degrees in sports sciences, especially the ones enrolled in some itineraries. However, even with this evolution during the degree, their anti-doping knowledge was modest. From a practical standpoint, previous research indicated that combining practical strength training with theoretical lessons was better at increasing knowledge of anabolic androgenic steroids than using theory alone in high school students [29]. Hence, the combination of theoretical lessons and practical classes about anti-doping may be one effective manner to increase anti-doping knowledge among university students of sports sciences.

The use of dietary supplements was also a variable that influenced the questionnaire score. Students who used dietary supplements scored higher in anti-doping knowledge compared to their counterparts who did not use dietary supplements. Data from numerous surveys [30] show that dietary supplement users are more likely than non-users to adopt positive health-related habits. This is also because dietary supplement users are habitually better educated and have higher incomes than nonusers. In sports, higher nutritional knowledge has been deemed as a protective factor against potential doping behaviors [31]. However, better nutritional knowledge or the use of dietary supplements are not factors directly associated with anti-doping behaviors. This is because nutritional supplements can be a source of unintentional doping as some supplements contain prohibited substances without showing this on their label [32]. Additionally, athletes who use doping (and their support personnel) often know more about anti-doping regulations and about dietary supplements than those who do not, as they need to be well-versed to dodge anti-doping measures. However, this does not entail that education *per se* is a double-edged sword. Doping and drug abuse in sports should be addressed with anti-doping education for athletes and with better-informed supporting personnel. In this regard, anti-doping education should be balanced by informing about the potential benefits of nutritional supplements but also about the risk associated with the use of supplements and drugs. The knowledge of the drawbacks and the criticism of using supplements in certain populations such as athletes younger than 18 years should be present even for some legal substances (e.g., caffeine [33] or creatine [34]). Among other issues, anti-doping education should provide tools for athletes and support personnel on how to assess the need, assess the risk, and assess the consequences of using dietary supplements [32]. On the other hand, education interventions should not use a “scare-based” approach (i.e., based upon negative health risks and fear appeals) as they are inefficient and may produce a boomerang effect while they should encompass a “knowledge-based” approach with information knowledge about drugs, specifically banned substances and prohibited methods, to improve efficacy [14,35]. Athlete support personnel should be aware of the pros and cons of dietary supplements to assist athletes to make an informed decision on supplement use. Anti-doping education is a primary aspect to reach this goal.

Another interesting finding is that students playing team sports had lower anti-doping knowledge than students playing individual sports. This result is consistent with another study showing that knowledge about doping among team athletes is low [36], while the percentage of adverse analytical findings is habitually lower in teams than in individual sports [37]. This information suggests that the anti-doping knowledge of university students undertaking degrees in sports sciences is influenced by factors unrelated to their studies at the university, such as dietary supplement use and the type of sport they practice. Although there are various influential social factors in sports, coaches undoubtedly play one of the most important roles in shaping the psychological experiences and behaviors of their athletes [38,39]. Students who will become coaches in the future will have a strong influence on young athletes because they habitually see coaches as one of their main sources of information [40]. Therefore, it is of vital importance that anti-doping education is fully covered in the syllabus of bachelor’s degrees in sports sciences to aid in preventing both intentional and inadvertent doping.

Our study has several limitations. First, we used a validated and reliable questionnaire based on the Play True Quiz developed by WADA with some modifications to be used for athlete support personnel. We used this questionnaire because there are no standardized investigative methods to assess anti-doping knowledge [41]. To date, this investigation is the first one using the modified version of the Play True Quiz, which limits the comparison of the results with previous studies. However, the authors intend to use this same questionnaire for future investigations with other populations of athlete support personnel. Second, although all 46 Spanish universities with bachelor’s degrees in sports sciences were invited to participate in this research, only responses from students from 26 universities were obtained. Additionally, the response rate was relatively low as, on average, only 39.2% of the students who were contacted finished the questionnaire. Although we obtained responses from 1233 students, it is still possible that the current study presents a partial view of the anti-doping knowledge of university students in sports sciences in Spain. Third, although the syllabus of degrees in sports sciences in all universities under investigation had a similar structure, they were not identical. Last, the current experiment includes a cross-sectional comparison of the anti-doping knowledge per course, as in previous investigations [25]. Future studies should include a cross-over approach to investigate how anti-doping knowledge evolves across the four courses of bachelor’s degrees in sports sciences.

## 5. Conclusions

The knowledge of basic anti-doping rules and doping prevention strategies of the bachelor students in sports sciences in Spain was suboptimal. This is because the scores obtained in the adapted version of the WADA Play True Quiz were modest despite this questionnaire included simple and basic questions about anti-doping. From a practical standpoint, it is not necessary for future graduates in sports sciences to become experts in legal regulations on doping, but it is essential that they understand the extent of doping and its negative consequences in medical, psychological, and sociological aspects of the athlete. Additionally, future graduates should possess skills to identify and prevent risky doping behaviors of the athletes with whom they will work. The results of this study represent a call for including specific anti-doping training in the syllabus of bachelor’s degrees in sports sciences in Spain. We advocate for the inclusion of an anti-doping subject, with at least 60 h and with the status of compulsory for all students, within the syllabus of the sports sciences degrees as an easy and direct measure to enhance anti-doping knowledge of bachelor’s students. A second alternative is the inclusion of anti-doping lessons within well-established subjects of a sports sciences degree syllabus (e.g., exercise physiology, sociology in sports, etc.). In the future, it will be necessary to examine the effects of different approaches to enhance anti-doping knowledge in university students pursuing sports-related university degrees.

## Figures and Tables

**Table 1:** Gender, course, itinerary, and type of sport practiced in a sample of 1233 bachelor students undertaking a degree in sports sciences of Spanish universities that were questioned about anti-doping knowledge.

Variable	N (Frequency)
Gender	
Male	907 (73.6%)
Female	322 (26.1%)
Non-binary	4 (0.3%)
Total	1233 (100%)
Course	
First	267 (21.7%)
Second	517 (41.9%)
Third	269 (21.8%)
Fourth	180 (14.6%)
Itinerary	
Sports performance	382 (31.0%)
Health	223 (18.1%)
None	104 (8.4%)
Sports management	88 (7.1%)
Physical education	397 (32.2%)
Other	39 (3.2%)
Sports practiced	
None	81 (6.6%)
Individual	665 (53.9%)
Team-based	487 (39.5)

## Data Availability

The data presented in this study are available on request from the corresponding author. The data are not publicly available due to legal restrictions.

## Appendix A. Adapted Version of the WADA Play True Quiz

**1. Question:** Athletes are ultimately responsible for what they swallow, inject, or apply to their bodies.

**Answer:** True

**2. Question:** Only athletes competing at the Olympics, Paralympics, and World Championships are subject to doping control.

**Answer:** False

**3. Question:** WADA stands for: World Anti-Doping Administration or World Anti-Doping Agency?

**Answer:** World Anti-Doping Agency

**4. Question:** The maximum number of times an athlete can be tested each year is?

a. 2

b. 5

c. 20

d. Unlimited

**Answer:** d. Unlimited

**5. Question:** The analysis of urine for the detection of prohibited substances or methods in sport can be performed by any laboratory with the necessary equipment?

**Answer:** False

**6. Question:** If a nutritional supplement is bought from a pharmacy (over-the-counter), it is definitely permitted in sports.

**Answer:** False

**7. Question:** When athletes are sick, they can be excused from taking any medicine to help them get well?

**Answer:** False

**8. Question:** A coach or doctor assisting or encouraging an athlete to take prohibited substances can be sanctioned if that athlete tests positive?

**Answer:** True

**9. Question:** In Spain, sports science graduates may act as doping control officers to collect urine samples at a competition if they complete the course that qualifies them for this function.

**Answer:** True

**10. Question:** Once the sample is collected and sealed and the paperwork is complete, any attempt to open, contaminate or otherwise tamper with the sample will be obvious.

**Answer:** True

**11. Question:** Athletes can refuse to submit to doping control if they are too busy.

**Answer:** False

**12. Question:** The prohibited list is different for each sport.

**Answer:** False

**13. Question:** The prohibited list identifies what substances and methods are prohibited in-competition and out-of-competition.

**Answer:** True

**14. Question:** How often is the prohibited list updated?

a. Once a month

b. Once a year, at least

c. Before every Olympic and Paralympic Games

d. It is never updated

**Answer:** b. Once a year, at least

**15. Question:** The “TUE” program provides athletes with the opportunity to request treatment for a serious medical condition by using a prohibited substance. What does TUE stands for?

**Answer:** a. Therapeutic use exemption

**16. Question:** According to the World Anti-Doping Code, the sanction for an athlete who has committed doping is 1 year.

**Answer:** False

**17. Question:** If a doping control officer comes to the athlete’s home to conduct an out-of-competition test, is it okay for him/her to leave the room alone to make a cup of coffee?

**Answer:** False

**18. Question:** If athletes have had an out-of-competition test already this week, it will be a few weeks before they have a new doping test.

**Answer:** False

**19. Question:** If the doping control officer does not have any identification, athletes can refuse to be tested.

**Answer:** True

**20. Question:** The person who receives the athlete’s sample at the laboratory received information about the name of the athlete.

**Answer:** False

**21. Question:** After athletes give a sample (blood and/or urine), for how long can it be stored and re-analyzed?

a. An indefinite period

b. 10 years

c. 2 years

d. It cannot be stored

**Answer:** b. 10 years

**22. Question:** A minor (<18 years) cannot be sanctioned for doping.

**Answer:** False

**23. Question**. Injured athletes cannot be tested for doping control tests.

**Answer:** False

**24. Question:** Athletes can be drug tested during a competition, even if they didn’t compete.

**Answer:** True

**25. Question:** If an athlete is tested positive for a prohibited substance, he/she has the right to:

a. Request the B sample be analyzed

b. Attend or to be represented for the opening and analysis of the B sample

c. Request copies of the laboratory documentation package

d. All of the above

**Answer:** d. All of the above

**26. Question:** When athletes are notified of doping control, do they need to report immediately to the doping control station?

a. Yes

b. No—They have one hour

c. They can report when they are ready

d. No—They have 24 h

**Answer:** a. Yes

**27. Question:** If athletes are banned from their sport, they can compete in another sport.

**Answer:** False

**28. Question:** If athletes test positive in their country, they can compete for another country.

**Answer:** False

**29. Question:** Can athletes be found to have committed an anti-doping rule violation (ADRV) if they consume a supplement that is contaminated with a prohibited substance?

**Answer:** Yes

**30. Question:** Who determines whether your application for a therapeutic use exemption (TUE), allowing you to use a prohibited substance for medical necessity, is approved or denied?

a. A committee of athletes

b. A group of professionals working for the national/international sports federation

c. A committee of medical experts

d. The president of the national or international sports federation

**Answer:** c. A committee of medical experts

**31. Question:** If a prohibited substance is found in an athlete’s backpack, the athlete cannot be sanctioned (it is necessary to prove that the substance is in the athlete’s body)

**Answer:** False

**32. Question:** The Athlete Biological Passport (ABP) is an anti-doping method to check that athletes have the same level of fitness throughout the season.

**Answer:** False

**33. Question:** Even if athletes are injured and not competing, they still need to submit their whereabouts information to the relevant sporting bodies in case they need to locate them for a drug test.

**Answer:** True

**34. Question:** A positive test is the only way an athlete can be sanctioned.

**Answer:** False

**35. Question:** Athletes can be sanctioned for associating with a coach, physician, or other such support personnel who are serving a period of ineligibility due to an anti-doping rule violation (ADRV).

**Answer:** True

**36. Question:** Substances included in the prohibited list are those substances that increase sports performance, also called ergogenic aids.

**Answer:** False

**37. Question:** Which of these apps makes it easy and accessible to check whether a medicine in Spain contains a banned substance?

a. NoDopApp

b. AppMed

c. Medicine App

**Answer:** a. NoDopApp
